# The Chemical Analysis and Physiologic Experiments on “Meatox,” the New Food Product

**Published:** 1907-12

**Authors:** I. V. S. Stanislaus

**Affiliations:** Medico-Chirurgical College, Philadelphia, Department of Pharmacy, Dean’s Office


### ABSTRACTS.

### The Chemical Analysis and Physiologic Experiments on "Meatox," the New Food Product.

By I. V. S. STANISLAUS, B.Sc., Pharm. D.
Medico-Chirurgical College, Philadelphia, Department of Pharmacy, Dean's Office.

[Abstract of an Original Communication published by *The Medical Bulletin* of Philadelphia, Pa., September, 1907.]

August 23, 1907.

*Editor Medical Bulletin, Philadelphia*:

PHYSIOLOGICAL EXPERIMENTS ON MEATOX.

Dear Sir: Enclosed please find the report of the chemical analysis made by me and of physiologic experiments conducted by me upon "meatox," a food product recently introduced by Mr. Charles Marchand, the well-known New York chemist.

"Meatox," as will be seen from below given findings, is in a class by itself. It can hardly be called a "meat extract" in the ordinary sense of the word, as it represents *more food-value* than any of these. The findings given below speak for themselves.

Very respectfully,

I. V. STANLEY STANISLAUS.

Philadelphia, Pa., March 28, 1907.

ANALYSIS OF MEATOX.

Upon analysis of a sample of meatox it was found to be composed of the following ingredients and proportions:

| | |
|---|---|
| Moisture | 4.80 |
| Celery Flavoring (residue from alcohol extract) | 2.21 |
| Sodium Chlorid | 4.56 |
| Proteid Matter | 73.54 |
| Insoluble Matter | 9.43 |
| Total | 94.54 |
| Ash | 4.96 |
| | 99.50 |
| Loss | .50 |
| | 100.00 |

PHYSIOLOGICAL EXPERIMENTS ON MEATOX.

Submitting the enclosed analysis I take pleasure in stating that basing it on the protein contents this is the most wonderful exponent of the modern nutrients extant. It is practically five times the meat value as a food, and as such will command the attention of every physiologist and hygienist interested in food products.

Philadelphia, June 29, 1907.

Meatox is a pale yellow, granular powder, having the odor and taste of celery, with which it is flavored.

Its composition according to my analysis of March 28, 1907, is as follows: 73.54 per cent. of proteids, 4.5 per cent. of salts and 12 per cent. of fats.

It does not dissolve in cold water, but imparts to the water an opaque color. When applied in a solution to the frog's heart it increases the rapidity of the beat for a short time, then makes the beat stronger.

The effect on blood pressure in the human subject was noted. A man was fed on meatox, bread and butter, water, a little hot tea, without milk, and fruits and berries with a little sugar for ten days. Blood pressure was estimated with a Rivi-Rocci sphygmomanameter, the systolic pressure being only recorded. Before starting in on the meatox diet, the radial systolic pressure was 115 millimeters of mercury, while at the close of the diet the radial systolic pressure was 118 millimeters of mercury.

The effect of meatox was tried on intestinal peristalsis. The effect was studied after the method of Professor Magnus, of Heidelberg. A piece of the small intestines of about 10 centimeters in length was taken from a rabbit. This was placed vertically in a glass containing about 225 cubic centimeters of a modified Ringer fluid kept to the temperature of 37.5° C. One end of the intestine was attached to an L-shaped glass rod by means of an S-shaped pin hook, and the other end to a Porter's heart lever slightly weighted.

Oxygen was allowed to pass through the solution all the time. Before the meatox was added contractions were recorded, to get about as near normal contractions as possible. About 2 decigrams of meatox were then added, and it was noted that the tonus of the contractions increased but the frequency remained about the same. Larger doses of meatox were added to the intestine the frequency became a little slower, but the tonus and force of contractions increased markedly.

The recording of the effect of a maximal break electric shock was noted on a smoked drum by means of a Porter's muscle lever and moist chamber. The muscles contracted very rapidly. Both the systole and diastole were very rapid, and when a strong tetanising current was added it was noted that the contractions became stronger after the current was on for a few seconds. Return to the normal was gradual. From this it appears to assist in muscular contraction.

The above deductions convince that meatox as a concentrated food is an easily digested, assimilated, and sustaining condiment. It is the first in the field of foods as a true highly-organised proteid food. Acknowledgment is here made of the valuable assistance rendered by Joseph F. Ulman, M. D.

